# Spinal Epidural Hematoma After Accidental Epidural Catheter Removal in a Patient With Sepsis

**DOI:** 10.1002/ccr3.71158

**Published:** 2025-10-04

**Authors:** Keisuke Omiya, Takashi Matsukawa

**Affiliations:** ^1^ Department of Anesthesiology University of Yamanashi Chuo Yamanashi Japan

**Keywords:** accidental epidural catheter removal, blood clot, septic patient, spinal epidural hematoma

## Abstract

We present a case of spinal epidural hematoma after accidental epidural catheter removal. The findings highlight that a blood clot might have formed at the tip of the epidural catheter left without continuous administration. When accidental epidural catheter removal occurred, pulling of the blood clot might induce spinal epidural hematoma.

## 1 Case Presentation

Spinal epidural hematoma can occur during epidural catheter placement or removal. The incidence of spinal epidural hematoma has been reported in 2–20 cases per 100,000 epidural procedures [1, 2, 3]. Rapid diagnosis is recommended using T2‐weighted magnetic resonance imaging (MRI). Here, we report the case of a patient who had been initiated on continuous hemodiafiltration (CHDF) due to postoperative sepsis, disseminated intravascular coagulation (DIC), and acute kidney injury who developed spinal epidural hematoma after accidental epidural catheter removal.

**FIGURE 1:**
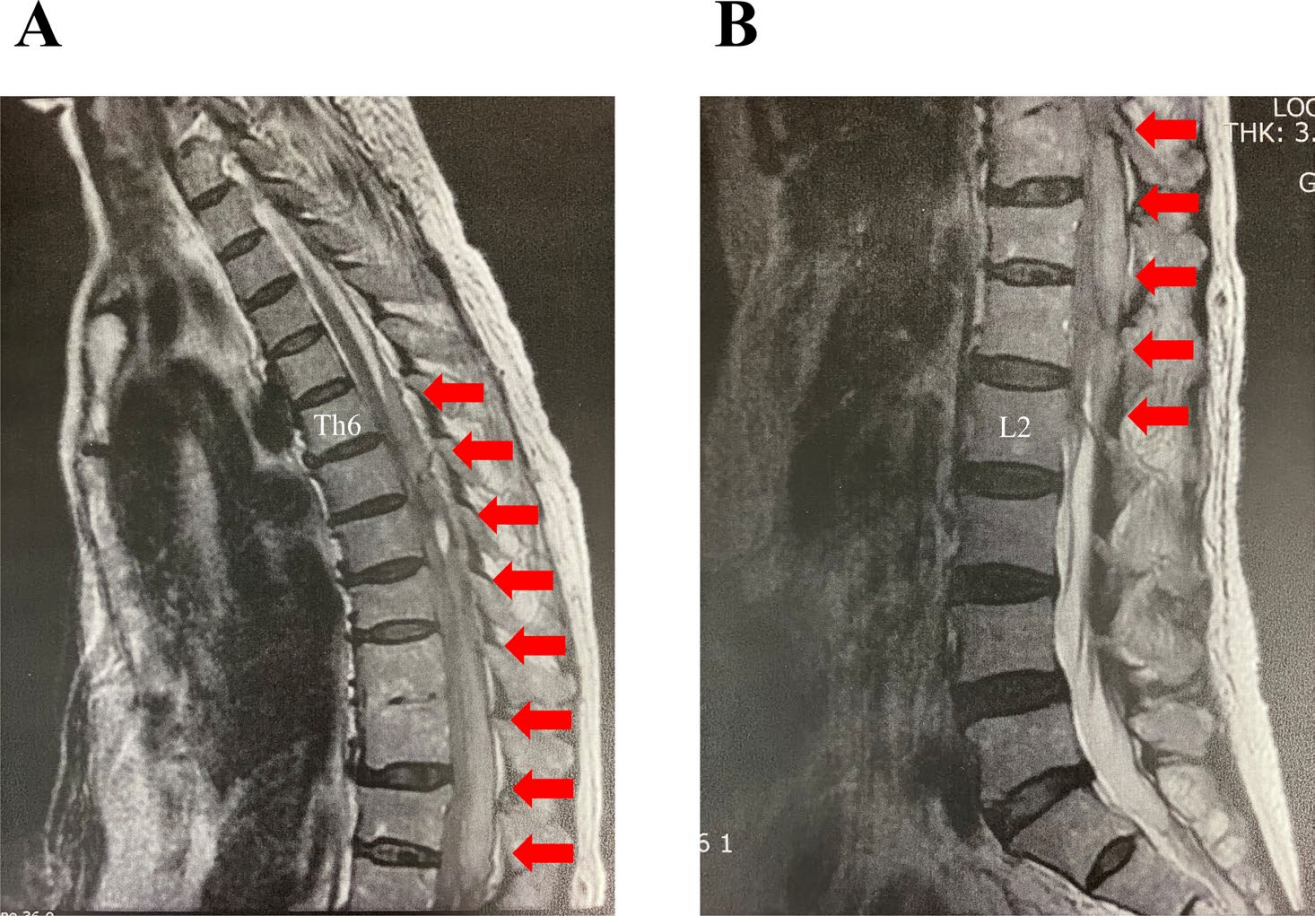
T2‐weighted sagittal magnetic resonance image. (A) Thoracic area; (B) lumbar area. Red arrows indicate spinal epidural hematoma from Th6 to L2.

A 67‐year‐old woman (height 154 cm; weight 60 kg) had a medical history of rectal cancer. Staging laparoscopy and cytoreductive surgery were scheduled for peritoneal dissemination. General epidural anesthesia was planned because the patient had undergone multiple laparotomies. No coagulopathy was observed preoperatively. The platelet count was 12.3 × 10^4^/μL (reference range 15.8–34.8 × 10^4^/μL). Prothrombin time (PT) was 93.0% (reference range 78.7%–123.1%) and activated partial thromboplastin time (APTT) was 30.6 s (reference range: 27.0–39.5 s). An epidural catheter was inserted at the Th12–L1 level and advanced by 5 cm into the epidural space. No neurological symptoms or hemorrhages were observed. Anesthesia was maintained with propofol, rocuronium, remifentanil, and 0.375% ropivacaine from the epidural catheter. The patient was then extubated in the operating room. Patient‐controlled analgesia (PCA) (4 μg/mL fentanyl, 0.06% ropivacaine, and 20 μg/mL droperidol) was started at 4 mL/h from the epidural catheter. On postoperative day (POD) 2, the patient showed high fever, hypotension, and low platelet count (2.3 × 10^4^/μL), indicating septic shock and DIC. Continuous PCA from the epidural catheter was stopped; however, the epidural catheter could not be removed because of the DIC. Emergency peritoneal lavage was performed. The patient was transferred to the intensive care unit under intubation with CHDF for sepsis and acute kidney injury. On POD 10, the patient was weaned from CHDF and extubated. On POD 12, the kidney injury persisted; therefore, the patient underwent dialysis with nafamostat. On POD 13, bleeding was observed at the epidural catheter insertion site when the nurse changed the patient's position. The catheter was removed and astriction was performed for 15 min. The platelet count was 4.3 × 10^4^/μL, the PT was 57.2%, and the APTT was 52.4 s. On POD 14, the patient complained of bilateral leg paralysis. MRI revealed spinal epidural hematoma at the Th6–L2 level (Figure 1). Immediate surgical intervention was not performed because of the DIC. On POD 105, the patient was transferred for rehabilitation.

## 2 Discussion

We present a case of spinal epidural hematoma after accidental epidural catheter removal. The findings highlight that the timing of the catheter removal should be considered. Furthermore, a blood clot might have formed at the tip of the epidural catheter. When accidental epidural catheter removal occurred due to the patient's position change, pulling of the blood clot might induce spinal epidural hematoma. The pulling of the blood clot at the tip of the epidural catheter had a high possibility and was not determined. Since this case, we no longer leave epidural catheters without continuous administration and have begun to administer 0.1 mL/h through the epidural catheter to prevent blood clot formation. We hope this intervention will reduce the incidence of spinal epidural hematoma.

## Author Contributions

**Keisuke Omiya:** conceptualization, investigation, resources, writing – original draft. **Takashi Matsukawa:** conceptualization, writing – review and editing.

## Ethics Statement

This manuscript complies with the provisions of the 1995 Declaration of Helsinki (as revised in Brazil 2013).

## Consent

Written informed consent was obtained from the patient to publish this report in accordance with the journal's patient consent policy.

## Conflicts of Interest

The authors declare no conflicts of interest.

## Data Availability

The data supporting this study's findings are available from the corresponding author upon reasonable request.
